# 4-(1-Benzyl-1*H*-benzo[*d*]imidazol-2-yl)-4-oxo-2-butenoic Acid Derivatives: Design, Synthesis and Anti-HIV-1 Activity

**DOI:** 10.22037/ijpr.2020.114341.14803

**Published:** 2021

**Authors:** Nafiseh Karimi, Rouhollah Vahabpour Roudsari, Mahsa Azami Movahed, Zahra Hajimahdi, Afshin Zarghi

**Affiliations:** a *Department of Pharmaceutical Chemistry, School of Pharmacy, Shahid Beheshti University of Medical Sciences, Tehran, Iran. *; b *Student Research Committee, Department of Pharmaceutical Chemistry, School of Pharmacy, Shahid Beheshti University of Medical Sciences, Tehran, Iran. *; c *Department of Medical Lab Technology, School of Allied Medical Sciences of Shahid Beheshti University of Medical Sciences, Tehran, Iran. *

**Keywords:** Design, Synthesis, Integrase, Anti-HIV-1, 4-Oxo-2-butenoic Acid

## Abstract

Acquired immunodeficiency syndrome (AIDS) is still an incurable disease with increasing mortality rate. Despite the development of effective FDA-approved anti-HIV drugs, there are some problems due to the growing of resistant viral strands. Therefore, discovery of novel anti-HIV agents is so needed. Integrase, targeted in highly active antiretroviral therapy (HAART), is a crucial enzyme in viral replication. In this study, new benzimidazolyl diketo acid derivatives were designed according to required features for inhibitors of HIV-1 integrase. Designed compounds were synthesized and evaluated for anti-HIV-1 effects. According to the cell-based biological assay’s results, most of the tested compounds demonstrated good anti-HIV-1 activity, ranging from 40-90 µM concentration with no severe cytotoxicity. The most potent compound was **13g** with EC_50_ value of 40 µM and CC_50_ value of 550 µM. Docking analysis of compound **13g** in integrase active site was in good agreement with well-known integrase inhibitors, proposing that anti-HIV-1 potency of compounds may be via integrase inhibition.

## Introduction

Acquired immunodeficiency syndrome (AIDS), induced by human immunodeficiency virus (HIV), remains a devastating infectious disease all over the world. It has been estimated that approximately 36.7 million people are living with HIV-1 (1). During the past two decades, over 30 FDA-approved anti-HIV drugs have been developed with different mechanisms, such as targeting viral diffusion, reverse transcription, DNA polymerization, and integration (2). In recent years, progressions of HIV-1 infection have been managed by highly active antiretroviral therapy (HAART), in which a combination of various anti-HIV drugs is used (3). Integrase (IN), one of the critical enzymes in the virus life cycle, catalyzes an important process in viral replication. It inserts the product of the reverse transcription, viral double strand DNA (dsDNA), within the human genome. The integration step is composed of two reactions called 3′-processing (3′-P) and strand transfer (ST) that both of them are mediated by HIV-1 IN enzyme. The (3′-P) reaction is carried out in the host cell′s cytoplasm, in which IN hydrolyses a phosphodiester bond and removes a conserved GT dinucleotide from the 3′-end of the viral DNA releasing two free 3′-OH groups as nucleophiles. The package of reverse transcripted viral DNA and IN, preintegration complex (PIC), transfers into the nucleus where the next step, strand transfer, occurs. During the ST reaction, IN enzyme catalyzes the joining of virus DNA to the host DNA (4-8). 

**Figure 1:**
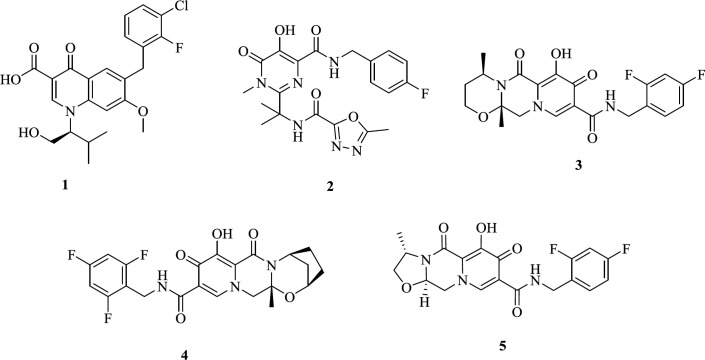
FDA-approved HIV-1 IN inhibitors, Elvitegravir (**1**), Raltegravir (**2**), Dolutegravir (**3**), Bictegravir (**4**), Cabotegravir (**5**).

IN has been considered as an appropriate target for the development of antiretroviral agents (9-11). To date, four HIV-1 INST inhibitors (INSTIs) have been developed that selectively suppress the ST reaction (12): The first generation Elvitegravir **(1)** and Raltegravir **(2)**, and the second generation Dolutegravir **(3)** and Bictegravir** (4)** (Figure 1) (13-19). These approved integrase inhibitors fit at the IN/DNA interface tightly and inhibit the HIV IN by binding to the Mg^2+^ ions present in the active site (20, 21). Resistance of HIV strain to the first generation is confirmed (22, 23); therefore, recently, many efforts have been made to develop a new generation of INSTIs to overcome the resistant mutant. Cabotegravir **(5)** is another generation integrase strand transfer inhibitor under clinical investigation (24, 25).

**Figure 2:**
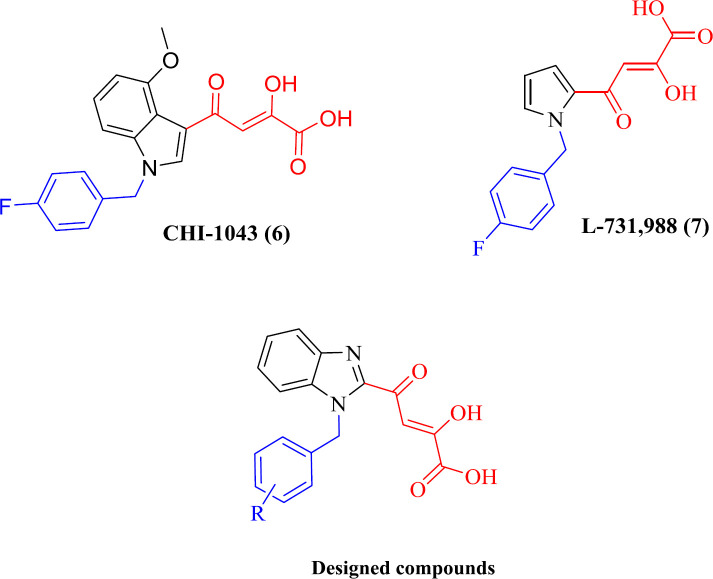
IN inhibitors (CHI-1043 (**6**), L-731,988 (**7**)) and designed compounds

Beta-Diketo acid (DKA) derivatives were identified as the first selective inhibitors of the IN strand transfer process. This compound class is characterized by a 4-oxo-2-butenoic acid moiety that is capable of coordinating Mg^2+^ ions and a flexible halobenzyl portion that interacts with the hydrophobic pocket present in the enzyme active site. Among DKA derivatives, indolyl and pyrrolyl diketo acids (exemplified by compounds CHI-1043 (**6**) and L-731,988 (**7**), respectively) exhibited submicromolar inhibitory activity in both enzymatic and cellular assays with low cell toxicity (26-30). Considering the above facts, and as part of our studies on IN inhibitors, we designed a novel series of DKA derivatives by replacing the central pyrrolyl or indolyl rings with the bicyclic benzimidazolyl platform (Figure 2). Different substituted benzyl groups were examined at the N-1 position of benzimidazolyl ring because the nature and pattern of substitution significantly affect the IN inhibitory activity (27, 28). 

## Experimental

*General*

All materials and solvents applied in this study were prepared from Merck AG and Aldrich Chemical. Melting points were determined with a Thomas–Hoover capillary apparatus. Infrared spectra were recorded using a Perkin Elmer Model 1420 spectrometer. ^1^HNMR and ^13^CNMR spectra were acquired on a Bruker FT-400 MHz instrument (Brucker Biosciences, USA). DMSO-*d*_6_ was used as a deuterated solvent. A 6410 Agilent LCMS triple quadrupole mass spectrometer (LCMS) with an electrospray ionization (ESI) interface was used to acquire the mass spectra. The standard methods were used to dry the organic solvents. Analytical results were determined within ± 0.4% of theoretical values. C, H, and N elemental analysis were performed on a Costech 4010 elemental analyzer. 

*Synthesis of compounds 1-(1H-benzo[d]imidazol-2-yl)ethan-1-ol (* ***10*** *) and 1-(1H-benzo[d]imidazol-2-yl)ethan-1-one (* ***11*** *)*

Compounds **10** and **11** were synthesized according to the procedures described in the literature (31). Melting point and spectroscopic data were compared with the literature for confirmation. 

*General Procedure for the Synthesis of* ***12a-i***

 To a solution of 1-(1*H*-benzo[*d*]imidazol-2-yl)ethan-1-one **11 **(3 mmol) in DMF at room temperature was added anhydrous K_2_CO_3_ (6 mmol). After stirring for 5 minutes, corresponding benzyl chlorides (3.3 mmol) were added drop wisely. The reaction mixture was stirred for 12 hours at room temperature. After completion, the mixture was poured into the ice water. Then the obtained precipitate was collected, filtered, and dried to give compounds **12a-i**. 

*1-(1-benzyl-1H-benzo[d]imidazol-2-yl) ethan-1-one (* ***12a*** *)*

Yield 98%; orange oil; IR (CHCl_3_) υ (cm^-1^) 1685 (C=O ketone), LC-MS (ESI) m/z 251 (M+H^+^).

*1-(1-(2-methylbenzyl)-1H-benzo[d]imidazol-2-yl)ethan-1-one (* ***12b*** *)*

 Yield 98%; orange oil; IR (CHCl_3_) υ (cm^-1^) 1695 (C=O ketone), LC-MS (ESI) m/z 265 (M+H^+^).

*1-(1-(3-methylbenzyl)-1H-benzo[d]imidazol-2-yl)ethan-1-one (* ***12c*** *)*

 Yield 98%; orange oil; IR (CHCl_3_) υ (cm^-1^) 1686 (C=O ketone), LC-MS (ESI) m/z 265 (M+H^+^).

*1-(1-(4-methylbenzyl)-1H-benzo[d]imidazol-2-yl)ethan-1-one (* ***12d*** *)*

 Yield 98%; orange oil; IR (CHCl_3_) υ (cm^-1^) 1674 (C=O ketone), LC-MS (ESI) m/z 265 (M+H^+^).

*1-(1-(2-chlorobenzyl)-1H-benzo[d]imidazol-2-yl)ethan-1-one (* ***12e*** *)*

 Yield 98%; orange oil; IR (CHCl_3_) υ (cm^-1^) 1677 (C=O ketone), LC-MS (ESI) m/z 285 (M+H^+^).

*1-(1-(3-chlorobenzyl)-1H-benzo[d]imidazol-2-yl)ethan-1-one (* ***12f*** *)*

 Yield 98%; orange oil; IR (CHCl_3_) υ (cm^-1^) 1686 (C=O ketone), LC-MS (ESI) m/z 285 (M+H^+^).

*1-(1-(2-fluorobenzyl)-1H-benzo[d]imidazol-2-yl)ethan-1-one (* ***12g*** *)*

 Yield 98%; orange oil; IR (CHCl_3_) υ (cm^-1^) 1688 (C=O ketone), LC-MS (ESI) m/z 269 (M+H^+^).

*1-(1-(3-fluorobenzyl)-1H-benzo[d]imidazol-2-yl)ethan-1-one (* ***12h*** *)*

 Yield 98%; orange oil; IR (CHCl_3_) υ (cm^-1^) 1683 (C=O ketone), LC-MS (ESI) m/z 269 (M+H^+^).

*1-(1-(3-methoxybenzyl)-1H-benzo[d]imidazol-2-yl)ethan-1-one (* ***12i*** *)*

 Yield 98%; orange oil; IR (CHCl_3_) υ (cm^-1^) 1687 (C=O ketone), LC-MS (ESI) m/z 281 (M+H^+^).

*General Procedure for the Synthesis of* ***13a-i***

To a stirring suspension of **12a-i **derivatives (2 mmol) in 10 mL dry diethyl ether at −10 °C was added diethyl oxalate (2.4 mmol). After stirring under an argon atmosphere for 10 min at −10 °C, a solution of sodium ethoxide (3 mmol) in 5 mL anhydrous absolute ethanol was added dropwisely. The reaction mixture was stirred at room temperature for 2 h. Then the organic phase was evaporated under reduced pressure, the obtained residue was dissolved in water. After acidification of the solution with 6M HCl solution to pH 2, the precipitates of DKA derivatives **13a-i** were yielded. The obtained solid was washed with water and methanol to afford pure **13a-i **products.

*4-(1-benzyl-1H-benzo[d]imidazol-2-yl)-2-hydroxy-4-oxo-2-butenoic acid (* ***13a*** *)*

Yield 50-60%; 127 °C. IR (KBr) υ (cm^-1^) 3414 (OH), 1722 (C=O acid), 1617 (C=O ketone). ^1^H NMR (DMSO-*d*_6_) δ 5.99 (s, 2H, N-CH_2_), 7.04 (br s, 1H, butanoate C3-H), 7.18-7.30 (m, 5H, benzyl H), 7.38-7.42 (t, 2H, J = 8 Hz, benzimidazol C5-H & C6-H), 7.70 (d, 1H, J = 6.8 Hz, benzimidazol C7-H), 7.87 (d, 1H, J = 6.4 Hz, benzimidazol C4-H); ^13^C-NMR (DMSO-*d*_6_) δ 48.58, 112.64, 121.38, 124.87, 126.91, 127.16, 127.98, 128.12, 129.13, 136.77, 137.40, 140.58, 145.85, 161.47, 162.06, 164.28; LC-MS (ESI) m/z 321 [M-H]^-^; Anal. (C_18_H_14_N_2_O_4_) C, 67.08; H, 4.38; N, 8.69; Found: C, 67.22; H, 4.15; N, 8.90.

*4-(1-(2-methylbenzyl)-1H-benzo[d]imidazol-2-yl)-2-hydroxy-4-oxo-2-butenoic acid (* ***13b*** *)*

Yield 50-60%; 142 °C. IR (KBr) υ (cm^-1^) 3460 (OH), 1740 (C=O acid), 1621 (C=O ketone). ^1^H NMR (DMSO-*d*_6_) δ 2.44 (s, 3H, CH_3_), 5.94 (s, 2H, N-CH_2_), 6.09 (d, 1H, J = 7.6 Hz, 2-methylbenzyl C6-H), 6.94-6.98 (t, 1H, J = 7.2 Hz, 2-methylbenzyl C4-H), 7.11-7.15 (t, 1H, J = 7.2 Hz, 2- methylbenzyl C5-H), 7.22 (s, 1H, butanoate C3-H), 7.25 (d, 1H, J = 7.2 Hz, 2- methylbenzyl C3-H), 7.45-7.46 (t, 2H, benzimidazol C5-H & C6-H), 7.62 (d, 1H, J = 6.8 Hz, benzimidazol C7-H), 7.97 (d, 1H, J = 7.2 Hz, benzimidazol C4-H), 14.07 (br s, 1H, COOH); ^13^C-NMR (DMSO-*d*_6_) δ 19.22, 46.91, 102.89, 112.52, 121.48, 124.05, 124.94, 126.63, 127.00, 127.38, 130.61, 135.24, 135.67, 137.06, 140.59, 146.14, 164.25, 184.08, 189.74; LC-MS (ESI) m/z 335 [M-H]^-^; Anal. (C_19_H_16_N_2_O_4_) C, 67.85; H, 4.80; N, 8.33; Found: C, 67.99; H, 4.65; N, 8.10.

*4-(1-(3-methylbenzyl)-1H-benzo[d]imidazol-2-yl)-2-hydroxy-4-oxo-2-butenoic acid (* ***13c*** *)*

 Yield 50-60%; 133 °C. IR (KBr) υ (cm^-1^) 3444 (OH), 1725 (C=O acid), 1621 (C=O ketone). ^1^H NMR (DMSO-*d*_6_) δ 2.23 (s, 3H, CH_3_), 5.96 (s, 2H, N-CH_2_), 6.93-7.20 (m, 5H, 3-methylbenzyl H and butanoate C3-H), 7.44 (s, 2H, benzimidazol C5-H & C6-H), 7.72 (s, 1H, benzimidazol C7-H), 7.92 (m, 1H, benzimidazol C4-H); ^13^C-NMR (DMSO-*d*_6_) δ 21.47, 48.55, 112.64, 121.34, 121.88, 124.17, 124.35, 124.81, 126.82, 127.69, 128.65, 129.04, 136.79, 137.37, 138.30, 140.59, 145.98, 164.38; LC-MS (ESI) m/z 335 [M-H]^-^; Anal. (C_19_H_16_N_2_O_4_) C, 67.85; H, 4.80; N, 8.33; Found: C, 67.60; H, 4.91; N, 8.03.

*4-(1-(4-methylbenzyl)-1H-benzo[d]imidazol-2-yl)-2-hydroxy-4-oxo-2-butenoic acid (* ***13d*** *)*

 Yield 50-60%; 146 °C. IR (KBr) υ (cm^-1^) 3417 (OH), 1729 (C=O acid), 1617 (C=O ketone). ^1^H NMR (DMSO-*d*_6_) δ 2.23 (s, 3H, CH_3_), 5.94 (s, 2H, N-CH_2_), 7.09 (m, 4H, 4-methylbenzyl H), 7.20 (s, 1H, butanoate C3-H), 7.40-7.43 (t, 1H, J = 6.8, benzimidazol C6-H), 7.45-7.49 (t, 1H, J = 7.6, benzimidazol C5-H), 7.75 (d, 1H, J = 8, benzimidazol C7-H), 7.91 (d, 1H, J = 8, benzimidazol C4-H) What about OH groups?; ^13^C-NMR (DMSO-*d*_6_) δ 21.09, 48.07, 112.49, 121.82, 121.86, 124.14, 126.40, 127.23, 127.34, 129.60, 137.12, 141.57, 164.25; LC-MS (ESI) m/z 335 [M-H]^-^; Anal. (C_19_H_16_N_2_O_4_) C, 67.85; H, 4.80; N, 8.33; Found: C, 68.05; H, 4.94; N, 8.10.

*4-(1-(2-chlorobenzyl)-1H-benzo[d]imidazol-2-yl)-2-hydroxy-4-oxo-2-butenoic acid (* ***13e*** *)*

 Yield 50-60%; 137 °C. IR (KBr) υ (cm^-1^) 3414 (OH), 1733 (C=O acid), 1617 (C=O ketone). ^1^H NMR (DMSO-*d*_6_) δ 6.02 (s, 2H, N-CH_2_), 6.36 (d, 1H, J = 7.6 Hz, 2-chlorobenzyl C6-H), 7.13-7.17 (t, 1H, J = 7.6 Hz, 2-chlorobenzyl C5-H), 7.22 (s, 1H, butanoate C3-H), 7.28-7.32 (t, 1H, J = 7.6 Hz, 2-chlorobenzyl C4-H), 7.43-7.49 (m, 2H, benzimidazol C5-H & C6-H), 7.55 (d, 1H, J = 8, 2-chlorobenzyl C3-H), 7.64 (d, 1H, J = 7.4 Hz, benzimidazol C7-H), 7.96 (d, 1H, J = 7.6, benzimidazol C4-H); ^13^C-NMR (DMSO-*d*_6_) δ 46.89, 112.24, 121.59, 123.91, 124.97, 126.78, 127.09, 128.12, 129.44, 129.96, 131.73, 134.89, 136.90, 140.73, 146.23, 164.22; LC-MS (ESI) m/z 355 [M-H]^-^; Anal. (C_18_H_13_ClN_2_O_4_) C, 60.60; H, 3.67; N, 7.85; Found: C, 68.02; H, 4.70; N, 8.10.

*4-(1-(3-chlorobenzyl)-1H-benzo[d]imidazol-2-yl)-2-hydroxy-4-oxo-2-butenoic acid (* ***13f*** *)*

 Yield 50-60%; 149 °C. IR (KBr) υ (cm^-1^) 3440 (OH), 1729 (C=O acid), 1617 (C=O ketone). ^1^H NMR (DMSO-*d*_6_) δ 6.00 (s, 2H, N-CH_2_), 7.10 (br s, 1H, 3-chlorobenzyl C6-H), 7.23 (s, 1H, butanoate C3-H), 7.33-7.34 (m, 3H, 3-chlorobenzyl C2-H & C4-H & C5-H), 7.42-7.46 (t, 1H, J = 7.2, benzimidazol C6-H), 7.48-7.51 (t, 1H, J = 7.6, benzimidazol C5-H), 7.77 (d, 1H, J = 8, benzimidazol C7-H), 7.94 (d, 1H, J = 8, benzimidazol C4-H); ^13^C-NMR (DMSO-*d*_6_) δ 48.06, 112.48, 121.47, 124.96, 125.75, 125.88, 127.10, 128.00, 131.07, 133.74, 136.69, 139.93, 140.57, 145.86, 164.30, 184.03; LC-MS (ESI) m/z 355 [M-H]^-^; Anal. (C_18_H_13_ClN_2_O_4_) C, 60.60; H, 3.67; N, 7.85; Found: 60.52; H, 3.50; N, 7.55.

*4-(1-(2-fluorobenzyl)-1H-benzo[d]imidazol-2-yl)-2-hydroxy-4-oxo-2-butenoic acid (* ***13g*** *)*

 Yield 50-60%; 135 °C. IR (KBr) υ (cm^-1^) 3414 (OH), 1725 (C=O acid), 1621 (C=O ketone). ^1^H NMR (DMSO-*d*_6_) δ 6.05 (s, 2H, N-CH_2_), ), 6.75-6.79 (t, 1H, J = 7.6 Hz, 2-fluorobenzyl C5-H), 7.05-7.08 (t, 1H, J = 7.6 Hz, 2-fluorobenzyl C4-H), 7.23 (s, 1H, butanoate C3-H), 7.25-7.33 (m, 2H, 2-fluorobenzyl C3-H & C6-H), 7.42-7.46 (t, 1H, J = 7.2, benzimidazol C6-H), 7.47-7.51 (t, 1H, J = 8, benzimidazol C5-H), 7.73 (d, 1H, J = 8.4 Hz, benzimidazol C7-H), 7.94 (d, 1H, J = 7.6, benzimidazol C4-H); ^13^C-NMR (DMSO-*d*_6_) δ 43.16, 43.20, 112.34, 115.82, 116.03, 121.53, 124.37, 124.52, 124.93, 125.20, 125.23, 127.04, 128.32, 128.36, 129.97, 130.05, 136.88, 140.61, 145.97, 158.91, 161.34, 164.22, 184.26; LC-MS (ESI) m/z 339 [M-H]^-^; Anal. (C_18_H_13_FN_2_O_4_) C, 63.53; H, 3.85; N, 8.23; Found: C, 63.38; H, 3.61; N, 8.45.

*4-(1-(3-fluorobenzyl)-1H-benzo[d]imidazol-2-yl)-2-hydroxy-4-oxo-2-butenoic acid (* ***13h*** *)*

 Yield 50-60%; 144 °C. IR (KBr) υ (cm^-1^) 3406 (OH), 1725 (C=O acid), 1613 (C=O ketone). ^1^H NMR (DMSO-*d*_6_) δ 6.00 (s, 2H, N-CH_2_), 6.98 (d, 1H, 3-fluorobenzyl C6-H), 7.07-7.12 (m, 2H, 3-fluorobenzyl C2-H & C4-H), 7.22 (s, 1H, butanoate C3-H), 7.32-7.38 (m, 1H, 3-fluorobenzyl C5-H), 7.41-7.45 (t, 1H, J = 7.6 Hz, benzimidazol C6-H), 7.47-7.51 (t, 1H, J = 8, benzimidazol C5-H), 7.76 (d, 1H, J = 8.4 Hz, benzimidazol C7-H), 7.93 (d, 1H, J = 8, benzimidazol C4-H); ^13^C-NMR (DMSO-*d*_6_) δ 48.13, 112.50, 114.08, 114.30, 114.74, 114.94, 121.44, 123.10, 123.12, 124.93, 126.99, 131.16, 131.24, 136.70, 140.24, 140.31, 140.59, 145.88, 161.47, 162.06, 163.89, 164.29; LC-MS (ESI) m/z 339 [M-H]^-^; Anal. (C_18_H_13_FN_2_O_4_) C, 63.53; H, 3.85; N, 8.23; Found: C, 63.66; H, 3.62; N, 8.50.

*4-(1-(3-methoxybenzyl)-1H-benzo[d]imidazol-2-yl)-2-hydroxy-4-oxo-2-butenoic acid (* ***13i*** *)*

 Yield 50-60%; 130 °C. IR (KBr) υ (cm^-1^) 3414 (OH), 1729 (C=O acid), 1610 (C=O ketone). ^1^H NMR (DMSO-*d*_6_) δ 3.69 (s, 3H, OCH_3_), 5.96 (s, 2H, N-CH_2_), 6.67 (d, 1H, J = 7.6 Hz, 3-methoxybenzyl C6-H), 6.80-6.83 (m, 3H, methoxybenzyl C2-H & C4-H & C5-H), 7.21 (s, 1H, butanoate C3-H), 7.40-7.44 (t, 1H, J = 7.2 Hz, benzimidazol C6-H), 7.46-7.49 (t, 1H, J = 7.6 Hz, benzimidazol C5-H), 7.75 (d, 1H, J = 8, benzimidazol C7-H), 7.92 (d, 1H, J = 8, benzimidazol C4-H); ^13^C-NMR (DMSO-*d*_6_) δ 48.47, 55.44, 112.65, 112.96, 113.32, 119.09, 121.37, 124.87, 126.90, 130.31, 136.77, 138.95, 140.54, 145.87, 159.84, 164.29; LC-MS (ESI) m/z 351 [M-H]^-^; Anal. (C_19_H_16_N_2_O_5_) C, 64.77; H, 4.58; N, 7.95; Found: C, 64.53; H, 4.70; N, 8.12.

*In-vitro anti-HIV and cytotoxicity assays*

Anti-HIV-1 activity of synthesized compounds was determined by single cycle replication assay which was reported previously (32-34). We constructed plasmid containing the HIV-1 genome mutated in the *pol* gene, which was co-transfected with plasmids expressing the *pol* gene products reverse transcriptase (RT) and integrase (IN), and the glycoprotein G of vesicular stomatitis virus. The virions produced in HEK 293 T cells were antigenic, but able to replicate only for one cycle*, e.g.* first generation single-cycle replicable (SCR) virions. The compounds were dissolved in DMSO at different concentrations of 10 mmol/L to 10 μmol/L. These stocks were diluted 100 times in cell environment so that the final concentrations of compounds were 100 μmol/L to 10 nmol/L. Zidovudine (AZT) and DMSO (1% v/v) were used as positive and negative controls. All tests were performed in triplicate. In the presence of different concentrations of compounds, HeLa cells (6 × 10^3^ per well of 96-wells plate) were infected with single cycle replicable HIV NL4-3 virions (200 ng p24). The compounds were added to the cells’ environment simultaneously with viral infection. After 72 h of infection, the supernatants of cell culture were collected, and p24 antigen load was measured by capture ELISA (Biomerieux, France). Percentage inhibition of p24 expression in treated culture was calculated as inhibition rate of p24 (%). XTT proliferation method was performed to evaluate the cellular toxicity of compounds. XTT (sodium 3-[1 (phenyl aminocarbonyl)-3,4-tetrazolium]-bis(4-methoxy-6-nitro) benzene sulfonic acid) reagent was used according to the kit instruction (Roche, Germany). The cells were cultured in 96 well plates (3.5 × 10^4^ cells/well) containing fresh phenol-red free medium and incubated for 72 h in a CO_2_ incubator. Subsequently, 50 μL of XTT was added into each well and incubated 4 h at 37 °C. The plates were evaluated by ELISA reader at the test and reference OD of 450 and 630 nm, respectively. The cytotoxic concentration that reduced number of viable cells by 50% (CC50) was calculated after determining p24 load in the HIV-1 replication assay plates.

*Molecular modeling studies*

 Molecular modeling was performed using the Autodock Vina (35). 3OYA was used for binding mode analysis of HIV-2.54 cm inhibitory activity. The protein and ligands were prepared in Autodock tools 1.5.6 from MGL Tools package (36). The co-crystallized ligand and water molecules were extracted, Kollman charges were added, nonpolar hydrogens were merged, and AutoDock4 atom type was assigned to the protein structure. The ligand was created and minimized using HyperChem 8.0 (37). The active site was defined as a Grid box around the crystallographic ligand Raltegravir in 20 × 20 × 20 dimensions. Selected molecules were docked in the active site, and the bioactive conformations were generated using Autodock Vina.

## Results and Discussion

*Chemistry*

**Scheme 1:**
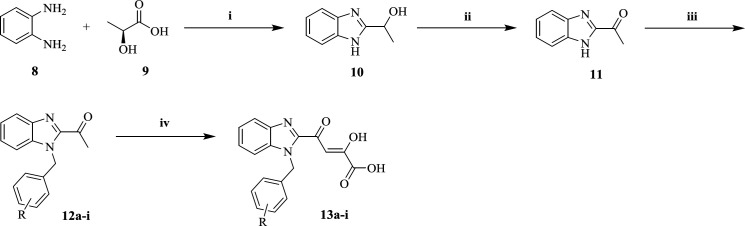
Reagents and conditions: (**i**) 4N HCl, reflax, 6h; (**ii**) K_2_Cr_2_O_7_, dil. H_2_SO_4_, rt; (**iii**) ArCH_2_Cl, K_2_CO_3_, DMF, rt, 12 h; (**iv**) diethyl oxalate, NaOEt/EtOH, diethyl ether, -10^ ○^C- 0 ^○^C, 2 h

 The preparation procedure of final benzimidazolyl DKA derivatives was shown in Scheme 1. 2-(α-hydroxyethyl)benzimidazole **10** was achieved via Philips condensation of commercially available *o*-phenylendiamine (**8**) and lactic acid (**9**) in 4N HCl (38). Then **10** was oxidized in the presence of K_2_Cr_2_O_7_ in dil. H_2_SO_4_ and the mixture was neutralized by aq.NH_3_ to pH of 5.5-6.0 to afford 2-acetylbenzimidazole **11 **intermediates (31). N-benzylation of **11** with substituted benzyl chlorides proceeded to yield **12a-i** derivatives (39), which were then condensed with diethyl oxalate using sodium ethoxide in diethyl ether and salt-ice bath to synthesis the corresponding DKA compounds **13a-i** (40).

*Anti-HIV-1 activities*

The novel benzimidazolyl DKA derivatives containing different substituted benzyl derivatives at N-1 position was synthesized and tested *in-vitro* for the inhibition of the single-cycle HIV-1 replication in HeLa cell culture. Raltegravir was used as a positive control. The synthesized compounds were also evaluated for cytotoxicity in a cell-based XTT assay. The biological results were expressed as anti-HIV-1 EC_50_, CC_50_, and SI (selectivity index, given by the CC_50_/EC_50_ ratio). The results of the biological activities of the synthesized compounds were summarized in Table 1. All newly synthesized compounds had a safety profile with no significant cytotoxicity (CC_50_ values > 380 μM). The tested compounds displayed EC_50_ values lower than 110 mM, except the compound **13e** with EC_50_ values of 155 μM. The compounds containing substituted benzyl moiety (**13b-i**) were more potent than unsubstituted corresponding compounds (**13a**). Among the compounds containing substituted benzyl moiety, increased activity was achieved when the substituents including fluoro, chloro, or methyl were introduced at the 2-, 3-, or 4-positions of benzyl ring, respectively (compounds **13g**, **13h**, **13f**, and **13d**). The compounds bearing 4-methoxybenzyl (**13i**) or 2- and 3-methylbenzyl groups (**13b** and **13c**) showed lower potencies. The best activity was observed for compound **13g** with EC_50_ value of 40 μM and SI value of 13.7.

*Molecular modeling*

**Figure 3:**
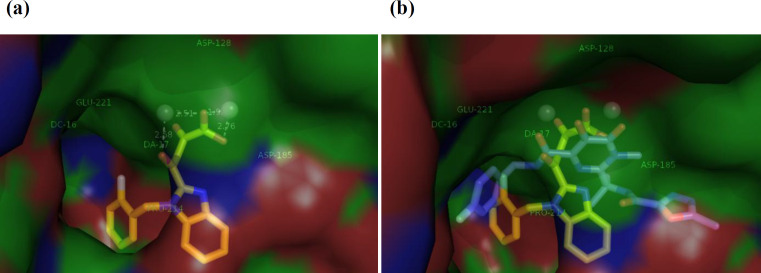
(a) The binding interaction of ligand **13g** (yellow) into the IN active site. IN active site and viral DNA strands are presented as surface. Amino acid residues (Asp128, Asp185, Glu221, Pro214) are determined with metal ions (Mg^2+^) in gray spheres. (b) Superposition of **13g** on the Raltegravir (blue) at IN binding site

 In order to examine the binding mechanism of our synthesized benzimidazolyl DKA derivatives, the most active compound **13g, **was selected for molecular docking studies. We performed molecular modeling studies by AutoDock Tools software using the X-ray structures of prototype foamy virus (PFV) intasome, an acceptable model for HIV integrase active site, in complex with double-strand DNA, two metal ions, and Raltegravir (PDB: 3OYA) (41, 42). The docking pose of compound **13g** within HIV IN CCD is illustrated in Figure 3a. According to the docking results, **13g** fits in the IN binding site perfectly through binding of a chelating triad in DKA with two Mg^+2^ ions present in the active site by the distance of 2.58, 2.51, 1.95, and 2.76 Å. On the other hand, based on the docking analysis, more confirmation is obtained by π-π stacking of 2-flourobenzyl group via placing in the hydrophobic pocket composed by viral nucleotide DA17 and DC16 and Pro214. Moreover, Figure 3b reveals that the binding mode of compound** 13g **resembles that of Raltegravir, suggesting that the tested compounds may exhibit the anti-HIV-1 activity through the inhibition of integrase enzyme.

**Table 1:** Cytotoxicity and Anti-HIV Activities of Compounds **13a-i**

		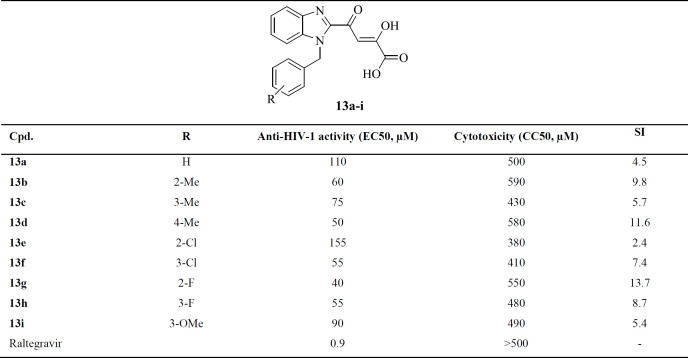		
**Cpd.**	**R**	**Anti-HIV-1 activity (EC50, ** **µM** **)**	**Cytotoxicity (CC50, ** **µM** **)**	**SI**
**13a**	H	110	500	4.5
**13b**	2-Me	60	590	9.8
**13c**	3-Me	75	430	5.7
**13d**	4-Me	50	580	11.6
**13e**	2-Cl	155	380	2.4
**13f**	3-Cl	55	410	7.4
**13g**	2-F	40	550	13.7
**13h**	3-F	55	480	8.7
**13i**	3-OMe	90	490	5.4
Raltegravir		0.9	>500	-

## Conclusion

 We have designed, synthesized, and evaluated a series of novel benzimidazolyl DKA derivatives bearing different substituted benzyl moiety for their inhibition of HIV-1 in cell culture. Most of the designed compounds demonstrated good to moderate potency with no significant cytotoxicity. The compound **13g** was found to be the most active in this series with An EC_50_ value of 40 μM and SI value of 13.7. Docking studies indicated that the binding mode of compound **13g** was similar to INSTIs. Therefore, benzimidazolyl DKA represents a potentially useful platform for further structural variations to find more potent anti-HIV-1 compounds. 
